# PolyQ 2.0: an improved version of PolyQ, a database of human polyglutamine proteins

**DOI:** 10.1093/database/baw021

**Published:** 2016-03-15

**Authors:** Chen Li, Jeremy Nagel, Steve Androulakis, Jiangning Song, Ashley M. Buckle

**Affiliations:** 1 Biomedicine Discovery Institute and Department of Biochemistry and Molecular Biology; 2 Monash Bioinformatics Platform, Monash University, Melbourne, Vic. 3800, Australia, and; 3 National Engineering Laboratory of Industrial Enzymes and Key Laboratory of Systems Microbial Biotechnology, Tianjin Institute of Industrial Biotechnology, Chinese Academy of Sciences, Tianjin 300308, China

## Abstract

Proteins with expanded polyglutamine (polyQ) repeats are involved in human neurodegenerative diseases, via a gain-of-function mechanism of neuronal toxicity involving protein conformational changes that result in the formation and deposition of β-sheet-rich aggregates. Aggregation is dependent on the context and properties of the host protein, such as domain context and location of the repeat tract. In order to explore this relationship in greater detail, here we describe PolyQ 2.0, an updated database that provides a comprehensive knowledgebase for human polyQ proteins. Compared with the previous PolyQ database, our new database provides a variety of substantial updates including detailed biological annotations and search options. Biological annotations in terms of domain context information, protein structural and functional annotation, single point mutations, predicted disordered regions, protein–protein interaction partners, metabolic/signaling pathways, post-translational modification sites and evolutionary information are made available. Several new database functionalities have also been provided, including search using multiple/combinatory keywords, and submission of new data entries. Also, several third-party plug-ins are employed to enhance data visualization in PolyQ 2.0. In PolyQ 2.0 the proteins are reclassified into 3 new categories and contain 9 reviewed disease-associated polyQ proteins, 105 reviewed non-disease polyQ proteins and 146 un-reviewed polyQ proteins (reviewed by UniProt curators). We envisage that this updated database will be a useful resource for functional and structural investigation of human polyQ proteins.

**Database URL:** http://lightning.med.monash.edu/polyq2/

## Introduction

The polyglutamine (polyQ) repeat containing proteins harbour a stretch of multiple consecutive glutamines (1). Expansion of the polyQ tract can lead to a toxic gain-of-function via a conformational change within the protein and the deposition of β-sheet-rich amyloid-like fibrils (2–4). As such, polyQ repeats are implicated in several neurodegenerative diseases, including Huntington disease and spinocerebellar ataxia (5–11). The length and domain context (i.e. the domains flanking the polyQ tract) is critical to the pathogenesis of the polyQ repeat (12–15). In addition to aggregation, other pathogenic mechanisms including calcium signaling (16), uncommon protein interaction (17) and proteasome dysfunction are also responsible for polyQ diseases (18–20). PolyQ 2.0 provides an improved tool to understand these diseases, and is a collection of all currently known human polyQ repeat-containing proteins. Nine of these proteins are implicated in pathogenesis, with the precise repeat threshold to pathogenesis varying within the disease subset (21–23).

Given the importance of polyQ repeats and their domain context information, we recently performed a bioinformatics investigation of the protein context of polyQ repeats (24), and constructed a web-accessible database of all human proteins containing a polyQ repeat greater than seven glutamines in length (25). Although the PolyQ database provides basic information for each entry, it lacks in both depth and breadth of annotation as well as functionality. Here, we present PolyQ 2.0, a substantially updated knowledgebase for human polyQ proteins. Compared with PolyQ, PolyQ 2.0 contains a variety of structural and functional annotations (such as polyQ protein disease models in mouse, protein 3D structure, Pfam domain, post-translational modification sites, single point mutations and complementary protein annotations), and domain context of polyQ repeats. Here, domain refers to a consecutive protein sequence motif. In addition, the usability of the web interface has also been improved, including the availability of database search with multiple keywords and user data submission with multiple levels of annotations including gene/protein basic information, protein structural and functional annotations. PolyQ 2.0 updates the MySQL relational database that stores entries, and enhances the web interface through the use of modern Javascript tools for visualization and interaction. Apache Tomcat mediates users’ access to the database through Java Servlets and JavaServer Pages.

### Update of database entries

**Figure 1.**
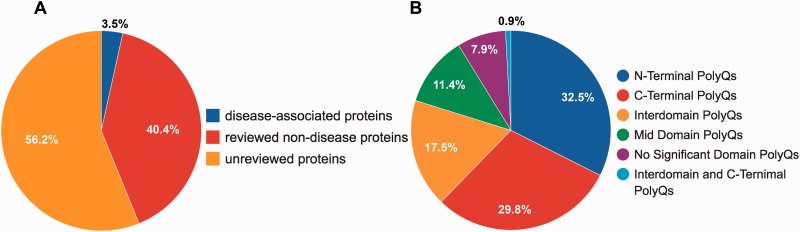
Statistics of data entries in PolyQ 2.0. (**A**) Distribution of disease-associated proteins, reviewed non-disease proteins and un-reviewed proteins. (**B**) Distribution of the sequence context of different types of polyQ domains for reviewed entries only.

Although PolyQ contained two types of polyQ proteins, namely disease and non-disease-associated, in PolyQ 2.0 all entries are categorized into three groups according to the annotation of disease involvement and completeness of review by UniProt curators. Here, disease-associated proteins refer to those proteins causing neurodegenerative diseases due to the abnormal expansion of polyQ repeats (e.g. Huntingtin; UniProt ID: P42858; PolyQ 2.0 ID: PD00043) rather than other proteins with common disease-associated mutations (e.g. CREB-binding protein; UniProt ID: Q92793; PolyQ 2.0 ID: PD00019). These groups are: reviewed disease-associated polyQ proteins, reviewed non-disease polyQ proteins and un-reviewed polyQ proteins. We first validated all the data entries in the previous PolyQ database with their UniProt annotation in order to ensure that only high quality data entries are included in PolyQ 2.0. Proteins were included as reviewed entries according to their annotation in the UniProt database. We incorporated polyQ proteins that have not been manually verified from UniProt as un-reviewed polyQ proteins for potential future reference. As a result, we obtained 9 reviewed disease-associated polyQ proteins, 105 reviewed non-disease polyQ proteins and 146 un-reviewed polyQ proteins (Figure 1A). We envisage that our identification and annotation of polyQ proteins in un-reviewed sequences may provide opportunities for disease discovery and further investigation. Since un-reviewed sequences are automatically annotated by UniProt but not manually verified, we will endeavor to keep entries updated as their annotation in UniProt evolves.

Following the classification system set out previously in PolyQ, we further classified all reviewed 114 sequences into 6 categories based on the locations and context of polyQ repeats relative to Pfam domains (26): (i) N-Terminal PolyQs—the first polyQ repeat appears before all Pfam domains (e.g. Ataxin-1; ID: PD00011), (ii) C-Terminal PolyQs—the last polyQ repeat appears after all the Pfam domains (e.g. CREB-binding protein; ID: PD00019), (iii) Interdomain PolyQs—the polyQ tracts appear between the first Pfam and last Pfam domain (e.g. BMP-2-inducible protein kinase; ID: PD00016), (iv) Mid Domain PolyQs—the polyQ repeat appears in the middle of a Pfam domain or overlaps with a Pfam domain (e.g. Atrophin-1; ID: PD00010), (v) No Significant Domain PolyQs—sequences that do not contain any significant Pfam domains (e.g. Mastermind-like domain-containing protein 1; ID: PD00053) and (vi) Unclassified PolyQs—sequences that do not fit into any of the above categories. The majority of polyQ domains are either N- or C-Terminal PolyQs, whereas only 7.9% of the reviewed polyQ containing entries do not harbor any significant Pfam domains (Figure 1B).

### Update of content and annotation

For PolyQ 2.0, the information content and annotations for entries have been significantly improved and expanded. The updated content includes basic protein information, protein structural information, predicted disordered regions, protein–protein interaction partners, metabolic/signaling pathways, single point disease- and non-disease associated mutations, and protein post-translational modification sites. In addition, we also performed BLAST search and generated multiple sequence alignments (MSAs) in order to provide evolutionary information for each protein entry. A comparison of protein annotations provided in PolyQ and PolyQ 2.0 is shown in Table 1.

**Table 1.** A comparison of protein annotation in PolyQ and PolyQ 2.0

**Content**	**PolyQ**	**PolyQ 2.0**
Gene information	No	Links to show overall gene information, gene sequences and variations
Protein information	Sequence and unstructured FASTA headers	Structured protein information (function, gene name, protein accession, etc.)
Protein 3D structure	No	Yes
Pfam domain	Yes	Yes
Protein disordered regions	No	Yes
Protein interaction partner	No	Yes
Metabolic/signaling pathway	No	Yes
Single point mutation	No	Yes, incorporating both disease-associated and nonsense mutations
Post-translational modification sites	No	Yes
Disease models on mouse	No	Yes
MSA	No	Yes

Annotations were extracted and reviewed from a variety of different publicly available resources, including ENA (27), 1000 Genome Project database (28), UniProt (29), Protein Data Bank (30), BioGrid (31), KEGG (32), SUPERFAMILY (33), Pfam (Version 27.0) (26) and Mouse polyQ database (34). We employed VSL2B (35) to annotate predicted disordered regions. Homologous sequence search was conducted using PSI-BLAST (36) (with an *E*-value of 0.001) against the UniProt database (http://www.uniprot.org/downloads). MSAs were generated using Clustal Omega (37). A summary of the database contents and annotations of PolyQ 2.0 and PolyQ is shown in Table 2.

**Table 2.** Summary of the database contents and annotations of PolyQ 2.0 and PolyQ

Annotation	PolyQ 2.0	PolyQ
Number of proteins	260	128
Number of protein structures	356	0
Number of protein interactions	4081	0
Number of single point mutations	704	0
Number of KEGG pathways	25	0
Number of Pfam domains	175	132
Number of post-translational modification sites	569	0

**Figure 2.**
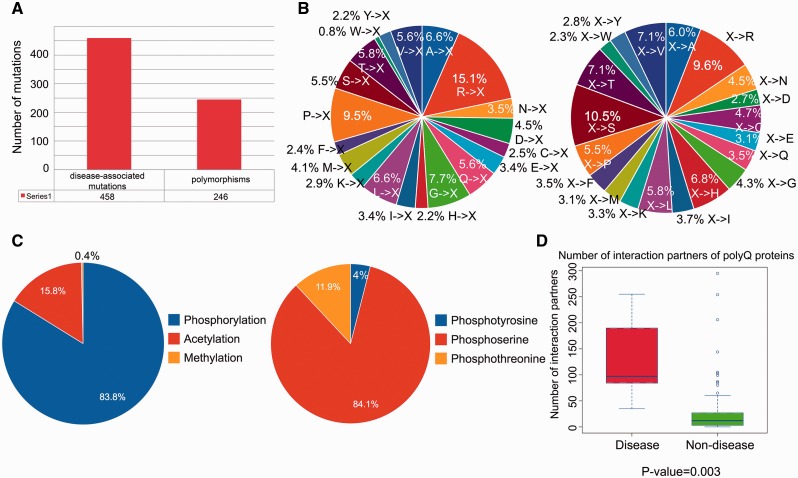
Statistical analysis of database content in terms of distributions of disease-associated mutations, post-translational modification site and number of protein–protein interaction partners. (**A**) Distribution of disease-associated mutation and polymorphism. (**B**) Distribution of the number of mutations with respect to two mutation patterns (where X means any amino acid). (**C**) Distribution of types of protein post-translational modification with detailed distribution of sub-types of phosphorylation. (**D**) Number of protein–protein interaction partners of reviewed polyQ disease-associated proteins and non-disease proteins.

We performed a statistical analysis of the database contents in terms of the distribution of disease-associated mutations, post-translational modification sites and number of protein–protein interaction partners. From a total of 704 single point mutations within the 114 reviewed entries, 458 (65.1%) mutations are disease-associated, whereas 246 (34.9%) mutations are polymorphisms (Figure 2A). We further defined two mutation patterns, i.e. *<A, C,…, V>→X* and *X→<A, C,…, V> *to identify trends in the wild-type amino acid that is mutated (Figure 2B, left) and the amino acid after mutation (Figure 2B, right). By analyzing the distribution of different types of mutations associated with polyQ proteins, we found that arginine is the most frequently mutated amino acid (accounting for approximately 15% of the mutated residues; Figure 2B, left). No trend was apparent for *X→<A, C,…, V> *type mutations (Figure 2B, right). Phosphorylation is the most frequently observed post-translational modification (Figure 2C). Disease-associated polyQ proteins have significantly more protein interaction partners than non-disease polyQ proteins (*P*-value  =  0.003; Figure 2D). However, interpretation of the biological relevance of these observations should be interpreted with caution as the dataset may contain bias towards disease-associated polyQ proteins due to their relatively greater interest compared with non-disease polyQ proteins. At the present time, these biological observations are based on the current dataset of PolyQ 2.0 and may vary when the database is updated.

### Database functionality and web interface improvements

PolyQ 2.0 features several important improvements of the user interface as well as new functionality, including database search with multiple types of keywords and new entry submission. A comparison of database functionality between PolyQ and PolyQ 2.0 is listed in Table 3.

**Table 3.** Database functionality comparison between PolyQ and PolyQ 2.0

**Functionality**	**PolyQ**	**PolyQ 2.0**
Database search		
Database ID/UniProt ID	No	Yes
Gene name	No	Yes
Protein name	Yes	Yes
Pfam domain	Yes	Yes
Disease	No	Yes
PTM	No	Yes
PTM kinase	No	Yes
Interaction partner	No	Yes
Combinatory search	No	Yes
User submission	No	Yes

**Figure 3.**
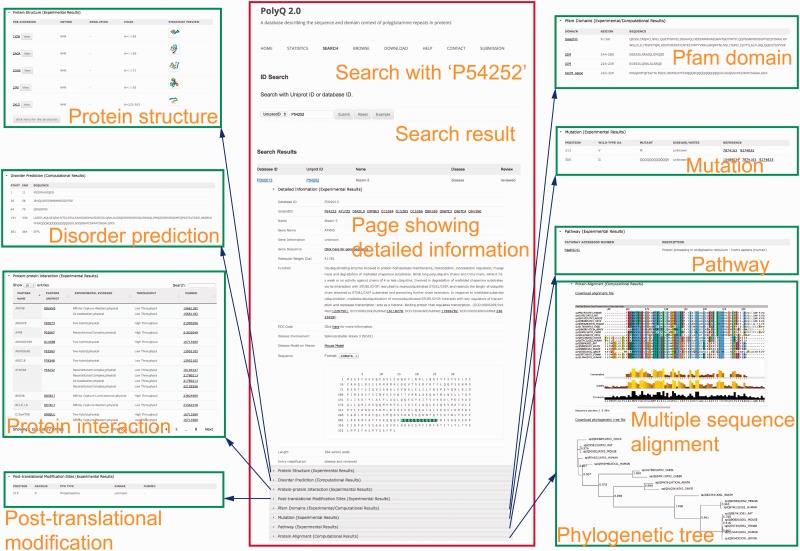
Typical search results in PolyQ 2.0 using the UniProt ID P54252 as an example. The website consists of nine sections showing detailed information for each entry, including gene/protein information, protein structure, metabolic/signalling pathway, protein interaction, post-translational modification site, Pfam domain, disorder region prediction, protein mutation and MSA.

The search functionality in PolyQ 2.0 has been considerably improved, with search options available using multiple keywords, in addition to the options of protein name and Pfam domain offered by the previous version. The database can be searched by PolyQ/UniProt ID, protein/gene name, Pfam domain, disease, type of protein post-translational modification sites/kinase, protein–protein interaction partner name, and combinatory keywords including disease, PTM type and polyQ domain context. The PolyQ ID is composed of ‘PD’ followed by five digits. As there are in total 260 entries in PolyQ 2.0, the PolyQ ID ranges from ‘PD00001’ to ‘PD00260’. An example of the result of database search with UniProt ID  =  P54252 (Ataxin-3) is shown in Figure 3, comprising nine main sections related to different annotations.

**Figure 4.**
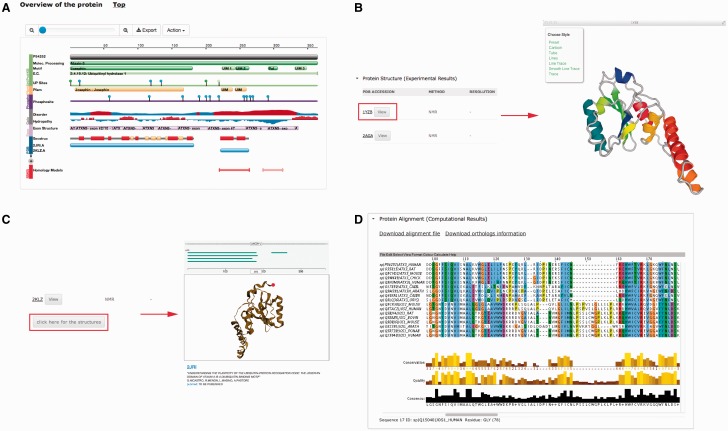
Plug-ins in PolyQ 2.0 to enhance database visualization. (**A**) Protein feature view plug-in. (**B**) PV showing protein structure. (**C**) pViz for visualizing multiple structures. (**D**) Jalview displaying MSAs.

Several plug-ins were employed to enhance visualization of database entries. In the protein basic information section, we embedded a protein feature view plug-in in order to show protein functional sites/domains and basic structural information (Figure 4A). PV (http://biasmv.github.io/pv/) and pViz (38) were also used to allow detailed examination of protein structures (Figure 4B and C). MSA is displayed using JalView (39) to visualize sequence conservation (Figure 4D). In addition to MSA, we generated the phylogenetic tree for each protein entry in PolyQ 2.0 using FastTree (40) and visualized the result using the jsPhyloSVG package (41). This provides a good visualization of the evolutionary relationships among the protein and its closest homologs. We also provided links to PhylomeDB (42) to show phylogenetic trees using a more comprehensive tree structure view.

Browsing of data entries has also been improved. The entries can now be categorized in terms of disease involvement and completeness of review and annotation. In addition, detailed context annotations, which show the distribution of polyQ domain, protein superfamily domain, Pfam domain and protein post-translational modification sites are available. The legend providing the explanations of the shapes and colours used can also be found at this page. In the figure provided, overlapped domains have been aligned horizontally based on their ranges. A webpage showing database statistics is available, giving users a one-page snapshot of database contents as well as convenient navigation around the database. Detailed user help and instructions are also provided. Finally, we made available a data submission page, where users can conveniently deposit their new polyQ sequences with detailed sequence/structural/functional annotations if applicable, including protein basic information, protein structural information, protein–protein interaction, mutation, functional domains/sites and pathway information. In addition, the submission for new entries does not require a specific format or style for the annotations. Users can provide simple sentences and references to describe the structural and functional annotations. All the submitted data will be formatted, structured and made publicly available once it has been carefully checked, curated and approved by a database administrator.

## Conclusions

Based on our previous PolyQ database for human polyQ proteins, in the present study we have developed an updated database, PolyQ 2.0, to provide comprehensive protein functional, structural and evolutional annotations together with domain context information for human polyQ proteins. Integrating publicly available annotations and computational resources, PolyQ 2.0 offers a variety of annotations in terms of protein basic information, protein structure, predicted intrinsically disordered domain, protein–protein interaction, protein functional site/domain, single point mutation, metabolic/signaling pathway and MSA. Given that the third-party databases (e.g. Pfam, UniProt, etc.) integrated in PolyQ 2.0 update regularly we will endeavor to update our database on a regular basis. This is particularly important since the categories of polyQ proteins defined in our study are based on the context information of polyQ and Pfam domains. In future revisions, we will cross-reference the NCBI Conserved Domains Database (43) to incorporate more functional domains into PolyQ 2.0 to enrich the annotations. We anticipate that this updated knowledgebase will benefit functional and structural studies of human polyQ proteins and their role in neurodegenerative diseases.
